# Amino acid composition predicts prion activity

**DOI:** 10.1371/journal.pcbi.1005465

**Published:** 2017-04-10

**Authors:** Fayyaz ul Amir Afsar Minhas, Eric D. Ross, Asa Ben-Hur

**Affiliations:** 1 Department of Computer and Information Sciences, Pakistan Institute of Engineering and Applied Sciences, Islamabad, Pakistan; 2 Department of Biochemistry and Molecular Biology, Colorado State University, Fort Collins, Colorado, United States of America; 3 Department of Computer Science, Colorado State University, Fort Collins, Colorado, United States of America; Indiana University, UNITED STATES

## Abstract

Many prion-forming proteins contain glutamine/asparagine (Q/N) rich domains, and there are conflicting opinions as to the role of primary sequence in their conversion to the prion form: is this phenomenon driven primarily by amino acid composition, or, as a recent computational analysis suggested, dependent on the presence of short sequence elements with high amyloid-forming potential. The argument for the importance of short sequence elements hinged on the relatively-high accuracy obtained using a method that utilizes a collection of length-six sequence elements with known amyloid-forming potential. We weigh in on this question and demonstrate that when those sequence elements are permuted, even higher accuracy is obtained; we also propose a novel multiple-instance machine learning method that uses sequence composition alone, and achieves better accuracy than all existing prion prediction approaches. While we expect there to be elements of primary sequence that affect the process, our experiments suggest that sequence composition alone is sufficient for predicting protein sequences that are likely to form prions. A web-server for the proposed method is available at http://faculty.pieas.edu.pk/fayyaz/prank.html, and the code for reproducing our experiments is available at http://doi.org/10.5281/zenodo.167136.

## Author summary

The determinants of prion formation in proteins that are rich in glutamine and asparagine are still under debate: is the process driven by primary sequence or by amino acid composition? In 2015 Sabate et al. published a paper suggesting that the process is triggered by short amyloid-prone sequences. Their argument was based on the success of their pWALTZ classifier, which uses a database of short peptides with known amyloid forming propensities. To explore the validity of their argument we compared their original scoring matrices with shuffled scoring matrices, and found no decrease in accuracy, suggesting that the success of pWALTZ is the result of the ability of the scoring matrices to capture amino acid composition. Furthermore, we propose a novel machine learning approach with accuracy that is superior to all published prion prediction methods that are currently available, and uses sequence composition alone.

## Introduction

Prion-forming proteins can exist in multiple structural states; in their prion state they form amyloid aggregates that are transmissible/infectious and are the cause of several diseases [1]. In mammals, conversion of the prion protein *PrP*^*Sc*^ to an amyloid form is toxic to cells and results in lethal neurodegenerative diseases like Creutzfeldt-Jacob disease, bovine spongiform encephalopathy, and kuru [2]. Furthermore, a number of recent papers have suggested that several common chronic disorders such as Alzheimer’s and Parkinson’s diseases as well as amyotrophic lateral sclerosis exhibit prion-like characteristics [3–5]. However, not all prions are harmful; in fact, it has been suggested that some prions may give selective advantage to individuals expressing these traits in certain environmental conditions [6].

Due to their unusual mode of inheritance and pathological significance, the study of prions is a very active area of research. Much of the research has been in the yeast *S. cerevisiae*, where prion-forming proteins are predominantly Q/N rich [7]. As the assays for confirming the prion activity of a protein are time consuming and very challenging to perform on a genome-wide scale, the development of methods for pre-screeing proteins for this function is an important task that can contribute to prion research in two major ways. First, such techniques can allow discovery of new prions and identification of their prion domains. A prion domain is the region within a prion protein that is essential for the protein to switch to a stable, heritable prion conformation [8, 9]. Second, such techniques can help identify the compositional, sequence, physiochemical or structural characteristics that are important for prion formation. In the research community there is no agreement on the primary forces that drive prion formation: whether it is guided mainly by amino-acid composition [9, 10], or as recent computational work suggests, that short sequences within Q/N rich disordered regions drive prion formation [11–13]. In what follows we present the experimental and computational findings related to this question.

The first two prion proteins discovered, Ure2 and Sup35 [14], both contain a Q/N-rich domain that is critical for prion activity [15–17], and scrambling the order of the amino acids in these prion domains does not inhibit prion formation [9, 10], highlighting a critical role for amino acid composition in driving prion activity. Various labs have developed methods to experimentally identify new prions based on amino acid composition. Early attempts focused primarily on identifying protein segments with high Q/N content [18–20]; these efforts contributed to the discovery of the prion protein Rnq1 [20] and the candidate prion New1 [21]. The first systematic study aimed at testing whether similarity in sequence composition is sufficient for distinguishing between prion and non-prion domains was performed by Alberti et al. [7]. They conducted a targeted proteome-wide search for promising prion candidates in *S. cerevisiae* using a Hidden Markov Model (HMM) trained on four known yeast prions. They then used four different assays to experimentally test the prionogenicity of the 100 top scoring protein domains. Out of these, a total of 18 domains were found to exhibit prion-like behavior in all four assays, while an additional 18 failed to show prion-like activity in any of the four assays. The availability of this experimental data opened the doors for development of more accurate methods for prediction of prion activity and understanding the nature of prion formation. Further experimental and computational work that supported the hypothesis that prion formation is primarily determined by sequence composition was performed by Toombs et al. [22]. They developed an *in vivo* method to quantitatively determine the prion propensity for each amino acid using a library of Sup35 mutants, and used these propensities as the basis for an algorithm called PAPA that predicts the prion-forming potential of a protein [23]. They demonstrated that PAPA, which uses sequence composition alone, is very effective at predicting prion activity in the dataset created by Alberti et al. [7]. Other work in this area such as that by Angarica et al. [24] and the Michelitsh-Weissman (MW) score [25] are also in agreement with these findings.

Recently several proteins with prion forming domains have been discovered in species other than yeast and human as a result of searches using PAPA and other methods. These include the bacterium *Clostridium botulinum* [26], Arabidopsis [27], and the fruit fly [28]. This suggests that prions are more prevalent than previously thought, and highlights the importance of accurate methods for identifying proteins with prion forming domains.

The recent model by Sabate et al. suggests that the presence of specific short amyloid-prone sequences that occur within intrinsically disordered Q/N rich regions are responsible for prion formation [13]. They measure the tendency of a Q/N rich region to be structurally disordered using the FoldIndex method and then score its propensity to form amyloids using WALTZ [29, 30]. For this purpose, WALTZ employs a position-specific scoring matrix (PSSM) developed from the amyloid properties of a set of hexapeptides. The resulting method, named pWALTZ, provides high accuracy for distinguishing between prions and non-prions from the Alberti dataset. Based on these results, Sabate et al. concluded that prion formation in Q/N rich yeast prions is predominantly driven by short sequence elements rather than its sequence composition.

The work presented here is a further discussion of the question of the extent to which primary sequence elements contribute to prion formation. We performed several computational experiments that provide support for the hypothesis that sequence composition is the primary driving force behind prion formation. First, we considered the argument of Sabate et al. that the high accuracy of pWALTZ suggests a role of primary sequence. In order to test this argument, we used *scrambled* PSSMs instead of those used in pWALTZ, and found no reduction in classification accuracy. Furthermore, we present a supervised machine learning algorithm called prion RANKing and classification (pRANK) that uses sequence composition alone, and has improved performance compared to all existing methods in predicting prionogenenicity of a protein sequence and localizaing its most prion-like domain. One of the major challenges in developing such a classifier is that the prion forming domain in a prion protein may either be unknown or its annotation may cover an area larger than the minimal set of amino acids required for prion formation. pRANK addresses this challenge through the use of multiple-instance learning (MIL) [31]. We demonstrate that multiple-instance learning allows us to model prion domain localization and classification very accurately using yeast and human data. Finally, we report results of *in silico* mutagenesis of non-prion sequences to increase prion activity and their correspondence with experimental findings published by Paul et al [32].

## Results and discussion

In this work we compare several methods designed for the prediction of protein priogenicity, i.e., whether a protein contains a prion forming domain, and demonstrate the effectiveness of using sequence composition alone for this task.

### Amino acid composition is the primary feature that predicts prion activity

Based on the accuracy of their pWALTZ prion prediction method, Sabate et al. have recently suggested a model of prion formation that depends on the presence of specific short sequence elements with high amyloid propensity [11]. On the basis of pWALTZ, which is designed to identify prions in Q/N rich proteins, the same group created PrionW—a web-server for the purpose of identifying prion-forming proteins at a genome-wide scale; PrionW first identifies proteins with disordered and Q/N rich regions, and proteins that pass these requirements are then scored by the pWALTZ scoring function [13]. The pWALTZ scoring function uses a Position Specific Scoring Matrix (PSSM) constructed from sequence with known amyloid propensity along with other features such as physiochemical properties and features based on structural modeling. In this work we investigate the contribution of primary sequence to the accuracy of pWALTZ/PrionW.

The PrionW PSSM is computed from hexapeptides with known amyloid potential. If short peptide sequence elements drive prion formation, then scrambling the PSSM with respect to position should reduce the accuracy of prion prediction (note that this form of scrambling is equivalent to scrambling the columns of the underlying multiple sequence alignment used to construct the PSSM). To test this, we generated all 720 possible permutations of the six columns in the scoring matrix and computed the average PSSM score across all permutations. We compared our implementation of pWALTZ-like scoring with PSSM features and its scrambled counterparts on two datasets. The first is a subset of the Q/N rich proteins tested by Alberti for which their assays indicated a clear distinction between prion-forming and non prion-forming domains [7]; the second dataset is designed to test the ability to detect prion forming proteins at a proteome-wide scale and consists of the Alberti proteins that are clear prion-forming as well as a non-redundant set of yeast proteins containing 5,575 sequences. Full details of the dataset are provided in the Methods section. If prions and non-prions can be distinguished due to primary-sequence properties rather than amino acid composition, scrambling the profile matrix in pWALTZ would result in a reduction of prediction accuracy. However, no decrease in performance is observed as shown in Table 1 for either of the two datasets. Similar results were obtained in two additional scrambling experiments. In the first, we scrambled the hexamers before forming the PSSM, again averaging over multiple permutations. In the second, we scrambled the prion domain, and scored it with the original PSSM. In both cases no decrease in accuracy over the orignal PSSM is observed (see Table 1). Finally, as shown next, we are able to predict prions with even higher accuracy using only amino acid composition features using a machine learning algorithm specifically designed to model prion-forming sequences.

**Table 1:** Accuracy of several variants of pWALTZ-like scoring. The method described as “original PSSM” uses the pWALTZ PSSM; “scrambled PSSM” averages over all permutations of the positions of the PSSM; “scrambled hexamers” first scrambles the hexamers from which the PSSM is constructed; “scrambled prion domain” tests the ability of the original PSSM to detect scrambled versions of the prion domain. Accuracy is measured using the area under the receiver operating characteristic curve (AUC-ROC) and the area under the precision recall curve (AUC-PR). All methods use a filtering step that considers only regions with negative FoldIndex scores and Q/N content of at least 25%. Figure A in S1 File provides the ROC and PR curves corresponding to these results, and Table A in S1 File provides additional results when no pre-filtering is performed.

pWALTZ-like method	Alberti dataset		Yeast proteome	
	AUC-ROC	AUC-PR	AUC-ROC	AUC-PR
original PSSM	87.4	91.3	96.4	12.3
scrambled PSSM	90.9	93.9	96.8	32.2
scrambled hexamers	88.4	92.0	96.6	22.4
scrambled prion domain	89.4	94.1	96.6	15.4

### Prion prediction with pRANK

One of the challenges in applying supervised learning to the problem of predicting protein priogenicity is that the labeled data—annotations of prion domains within a prion-forming protein—have inaccuracies that are the result of the difficulty of exact delineation of that domain. To address the uncertainty in the annotated prion domain we developed a method called prion RANKing and classification (pRANK) using the technique of multiple-instance learning (MIL), which allows to explicitly model this uncertainty [33, 34].

In what follows we compare various flavors of pRANK that use both position-dependent and position independent representations with the performance of several other methods: the HMM classifier of Alberti et al. [7], PAPA, and pWALTZ (Table 2). For this comparison, we have used the webserver implementation of pWALTZ called PrionW [13]. pRANK outperforms all other approaches in prion prediction, achieving a very high AUC-ROC score of 96.8% with PAPA is close second. Both methods use only amino acid composition, further evidence for the role of amino acid composition in prion formation.

**Table 2:** Classifier performance on the Alberti dataset. Performance is measured with leave-one-protein-out cross-validation using the area under the ROC curve (AUC-ROC) and the area under the precision recall curve (AUC-PR); the curves are provided in Figure B in S1 File.

Classifier	AUC-ROC	AUC-PR
pRANK	**96.8**	**96.8**
miSVM	92.2	90.4
SVM	87.4	87.8
Random Forests	88.0	90.6
PAPA	95.1	96.8
PrionW	86.7	89.8
PLAAC	68.7	74.7

In order to further explore the impact of primary sequence on prion prediction, we also experimented with a position dependent amino acid composition feature representation, which models a protein sequence as a vector of position-specific indicator variables. pRANK with position-dependent amino acid composition had an AUC-ROC score of 86.8%, much lower than the position-independent version. This low score provides further indirect evidence of the limited impact of primary sequence on prediction of prion domains.

Finally, we compared pRANK with the method of Angarica et al [24]. Angarica et al. report an AUC-ROC of 85% using a bootstrap performance evaluation protocol. Using the same bootstrap protocol, pRANK yielded an AUC-ROC of 92.30%.

### Localization of prion domains

pRANK can also predict the most prion-like domain within a protein by scoring each sequence window in a given protein. Our resuls show that pRANK provides very high accuracy for prion domain localization as well: the top pRANK prediction is always within the annotated prion domain for all proteins in our data set. For PAPA and prionW this is the case in only 86.4% and 72.7% of proteins, respectively.

### The role of multiple instance learning

**Fig 1:**
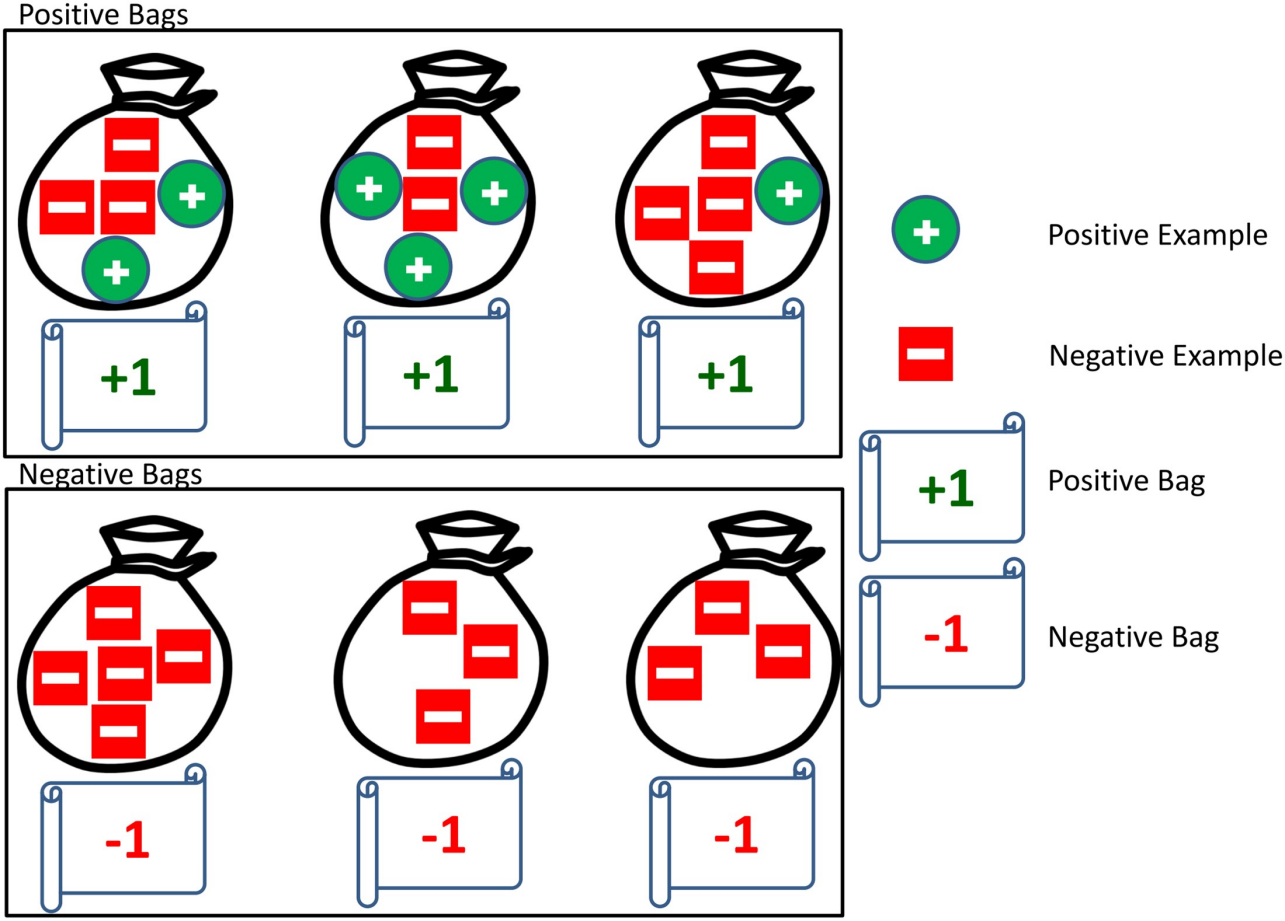
An illustration of the concept of multiple-instance learning. In MIL, training examples come in bags; a positive bag contains a set of examples, with the constraint that at least one of them must be positive. In our setup a positive bag corresponds to an annotated prion domain, and this constraint captures the inaccuracy that is inherent in experimentally delineating a prion-forming domain: the actual minimal domain that supports prion formation is rarely fully characterized, and is typically embedded within the annotated domain. The examples in a negative bag are all negative (all the sequence windows outside a prion-forming domain are negative examples).

To understand the improved performance of pRANK we need to understand the concept of MIL. In MIL, examples come in bags: positive and negative [33]. In our application an example is a window within a protein sequence; in a positive bag, a prion forming domain in this case, we assume that at least one sequence window in the bag is indeed positive; in a negative bag all examples are negative (see Fig 1). This is a weaker form of labeling positive examples, and captures the scenario in this classification problem very well, where not all of the sequence windows within an annotated prion domain are prionogenic. This allows the classifier to essentially ignore the windows that do not represent the target concept well. We have recently presented a novel formulation of MIL and demonstrated its advantage in detection of Calmodulin binding domains over several baseline approaches including standard multiple instance learning [34]. In this work we apply the same MIL formulation and present an improved training algorithm (see details in the Methods section).

To demonstrate the advantage of our MIL formulation, Table 2 compares the performance of pRANK with the accuracy of a regular support vector machine (SVM) and the multiple instance learning SVM (miSVM) of Andrews et al [33, 35]. pRANK outperforms the other SVM-based methods with an AUC-ROC of 96.8%. Furthermore, both multiple instance classifiers, miSVM and pRANK, perform better than a conventional SVM. This improvement in accuracy clearly indicates the usefulness of employing multiple instance learning in this problem. The conventional SVM does not model the ambiguity and noise in the experimental annotations, and is more affected by it than the MIL approaches. pRANK uses an ensemble of predictors (see Methods section for details); therefore we also compared its performance with Random Forests [36], which provided similar performance to the SVM classifier (see Table 2).

Other than an improvement in accuracy, pRANK offers several advantages over the miSVM formulation. miSVM requires optimization over the labels of examples in positive bags, making it a combinatorial optimization problem, which is solved using a heuristic approach based on iterative retraining an SVM with labels imputed in each iteration. As a consequence, miSVM may not converge to the optimal solution of the underlying mathematical formulation. Furthermore, the iterative re-training of an SVM in miSVM makes it very time consuming. Instead of constraints over labels, as in miSVM, the pRANK formulation involves constraints over the discriminant value of the highest scoring example in a positive bag (see Methods section). This is a more direct representation of the classification problem and leads to a better solution through stochastic subgradient optimization. Furthermore, it is a simpler model with fewer slack variables, which further contributes to the speed advantage of pRANK. pRANK takes under two minutes to train on the whole yeast proteome using a single core on an Intel i5 machine with 4GB of RAM whereas it was not feasible to run miSVM on this dataset on the same computer due to memory limitations.

### Evaluation over the yeast proteome

**Fig 2:**
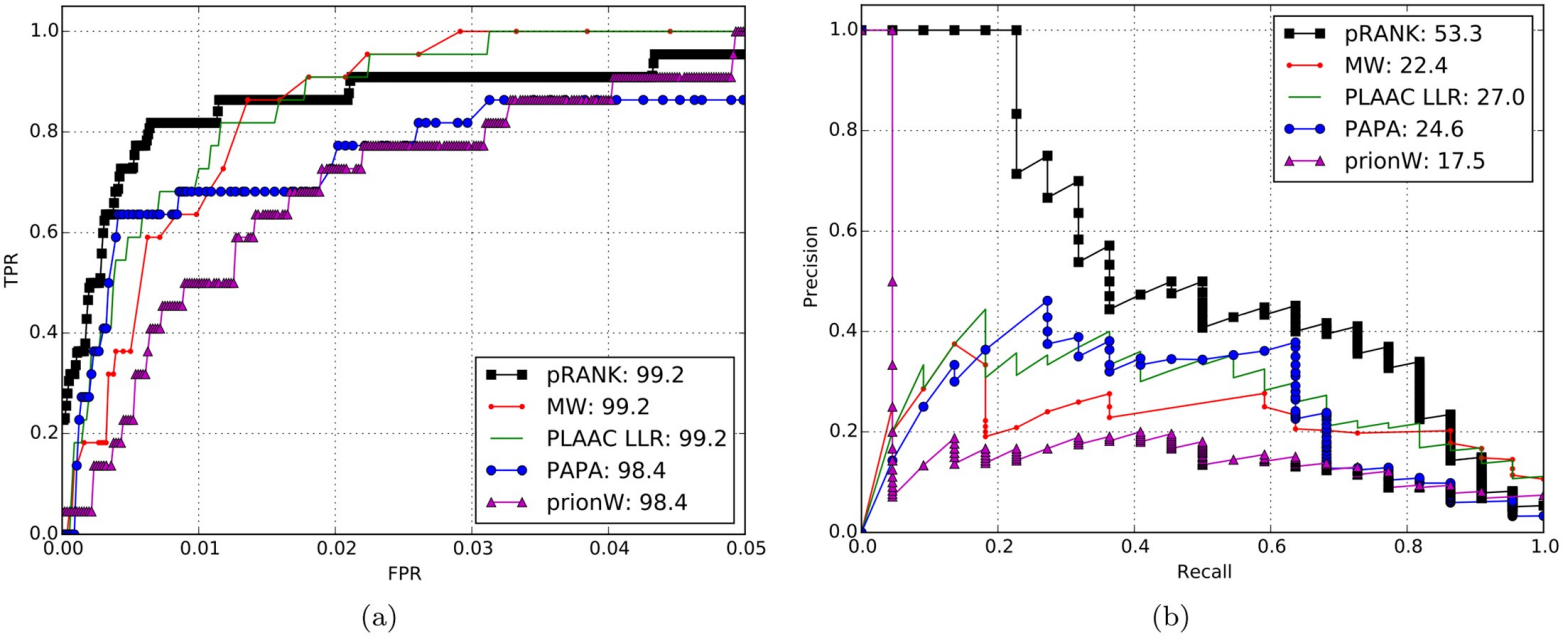
Receiver operating characteristic curves (a) and precision-recall curves (b) for proteome-wide prediction in yeast. FPR and TPR are the false and true positive rates. The numbers in parentheses represent the area under the curve. Notice that the x-axis of the ROC plot is trimmed at FPR of 5%. MW and PLAAC-LLR indicate the results for the Michelitsh-Weissman score and the HMM based algorithm presented in [25].

To compare the methods on a proteome-wide dataset we used all 22 known Q/N rich yeast prion domains and a non-redundant set of 5,575 yeast proteins as positive/negative examples. All proteins in the yeast proteome that were more than 90% similar to the known yeast prion proteins were removed; among the remaining proteins we ensured a level of sequence similarity of no more than 40%. In this analysis, we compared pRANK, PAPA, PrionW and two other methods: the Michelitsh-Weissman (MW) score and the PLAAC-Log Likelihood Ratio (LLR) [25]. The MW score is equal to the maximum number of Q or N residues in a sequence window with at least 80 amino acids. PLAAC-LLR is the HMM method used by Alberti et al., and then updated using additional prion-like domains [25]. The scores for these algorithms were obtained using the PLAAC webserver [25]. At the proteome level precision-recall curves are more informative than ROC curves due to only a small number of expected prions in the proteome. The PR curves in Fig 2 clearly show that pRANK outperforms all other methods at the proteome level as well: its AUC-PR score is 53.3%, while the next best method (PLAAC-LLR) has a score of 27.0%. We note that the score for PLAAC-LLR potentially overestimate its accuracy because some of the proteins in the yeast proteome have been used in training it. The advantage of pRANK can also be observed in the sharp initial rise in the ROC curves in Fig 2 compared to the other methods.

To obtain insight on the predictions made by pRANK we performed a Gene Ontology enrichment analysis using the GOrilla tool [37] on the ranking produced by pRANK (excluding all known yeast prions), and compared the enriched categories with those that we got for the known yeast prions. Fourteen out of the 22 proteins that contain prion domains are annotated as “nucleic acid binding”, with an FDR q-value of 0.04. This category is also highly significantly enriched in the top 200 predictions made by pRANK (FDR q-value of 4.28E-7). In fact, the top enriched categories are all related to nucleic acid binding or transcription factor activity. At the bottom of the list we find a few other types of enriched categories such as “zinc ion biding” with an FDR q-value of 3.18E-2 (see Tables B and C in S1 File for details).

### Evaluation in other species

A number of human proteins contain prion-like domains that compositionally resemble yeast prion domains. In recent years mutations in several of these have been linked to degenerative disorders such as ALS and frontotemporal dementia [38, 39]. These proteins include TAF15, EWSR1, hnRNPA1, FUS, hnRNPA2, TDP43, and TIA1. We evaluated the ability of pRANK and other methods to distinguish these seven proteins from a non-redundant set of 15,948 proteins not known to have prion-like activity (applying our non-redundancy filter to the human prion-like proteins yielded a subset of five of them). The results reported in Table 3 again show the effectiveness of pRANK compared to the other methods. We note that human prion-like proteins have somewhat different sequence characteristics than yeast prions, so the weaker performance of PAPA for example, which was specifically designed for yeast prions, is not surprising. The three top ranking proteins are associated with diseases characterized by amyloid deposits: hnRNP-UL1, the top ranking protein by pRANK was recently shown to be involved in amyotrophic lateral sclerosis (ALS) [40]; mutations in the PrD of our second prediction, HNRPDL, causes limb-girdle muscular dystrophy [41]; the 3rd prediction, TRNAU1AP, is also associated with ALS [42].

**Table 3:** Classifier performance proteome wide. Performance is measured with leave-one-protein-out cross-validation using the area under the ROC curve (AUC-ROC) and the area under the precision recall curve (AUC-PR).

Classifier	yeast		human	
	AUC-ROC	AUC-PR	AUC-ROC	AUC-PR
pRANK	99.2	53.3	99.7	26.2
MW	99.2	22.4	96.5	1.10
PLAAC-LLR	99.2	27.0	99.7	5.20
PAPA	98.4	24.6	99.4	5.50
prionW	98.4	17.5	98.6	1.00

It was recently discovered that the Rho Termination Factor in *Clostridium botulinum* acts as a prion [26]. It is the first bacterial prion discovered thus far, and was the top PAPA hit in the *Clostridium botulinum* proteome. Using pRANK trained on yeast prions we found it is ranked six out of 3678 proteins, and its prion domain was correctly identified.

#### pRANK performance in proteins with polyQ tracts

**Fig 3:**
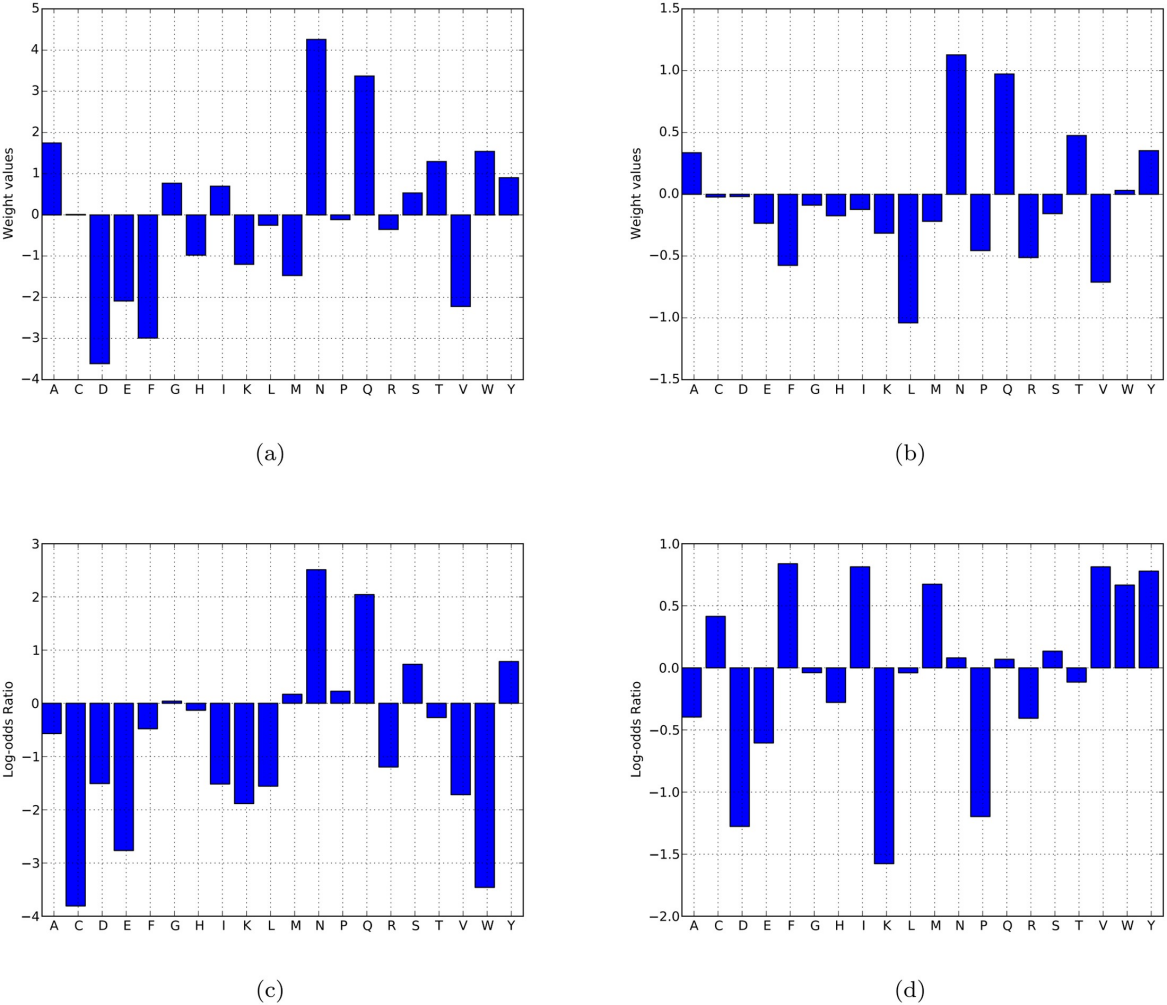
Comparison of amino acid weights for different methods. (a) the weights for pRANK over the Alberti dataset; (b) the pRANK weights in proteome wide evaluation; (c) shows the log-odds ratio, obtained by Angarica et al., of the frequencies of occurrence of different amino acids in prion domains in the yeast prions relative to their corresponding background frequencies in the protein universe. Figure (d) shows the log-odds ratios obtained experimentally by the random mutagenesis experiment by Toombs et al.

Since pRANK uses only amino acid composition, proteins with certain compositional biases can potentially show up as false positives. A prime example consists of proteins that contain a polyQ tract. Our non-redundant dataset of the yeast proteome contains 50 proteins with at least one occurrence of a polyQ tract of length 10. Because of the high weight assigned by pRANK to Q (see Fig 3), these can receive high scores from pRANK; however, the top 50 pRANK predictions contain only seven such proteins, and the median rank of these 50 proteins is 169. And interestingly, it has been shown that a polyQ domain can lead to aggregation in the presence of the prion form of Rnq1 [43]. Finally, we note that such false positives can arise for any classifier that uses amino acid composition exclusively, and the results need to be interpreted with care.

### Amino acid weights

To obtain an understanding of the importance of different amino acids for predicting priogenicity, we have plotted the weights assigned to individual amino acids by pRANK. These weights are plotted separately for the evaluations over the Alberti and proteome-wide data sets. For comparison, we have also plotted the log-odds ratios used in the methods by Angarica et al. and Toombs et al. (see Fig 3). First we note the high positive weights of Q and N as pRANK features in both pRANK evaluations. This correlates with the fact that all the prion domains in the Alberti dataset are Q/N rich and is also supported by the reported importance for these amino acids in the works by Angarica et al. and Sabate et al. These amino acids receive only a slightly positive score in the PAPA method, as it was specifically designed to answer a very different question, namely to predict how small compositional changes in a protein that resembles Sup35 affect its prion forming propensity. This observation also explains the lower performance of PAPA at the proteome level compared to pRANK.

Next, we observe that the two evaluations of pRANK produced somewhat different residue weights, which is a result of the fact that the task of priogenicity prediction in the Alberti dataset and the detection of prion proteins in a proteome are two different problems. The difference is the result in the choice of negative examples: The negative examples in the Alberti dataset are Q/N rich domains flagged by Alberti et al. as candidate prion domains, whereas they are random non-prions in the proteome-wide evaluation protocol. We note that when testing pRANK trained on Alberti negatives on a genome-wide test set we obtain an AUC-PR score of 30%, which is much lower than pRANK accuracy when trained genome-wide. This highlights the importance of choosing the negative examples to reflect the intended use of the classifier, i.e., use a wide selection of negative examples when using pRANK for a genome-wide screen.

We note a few differences between the log odds ratios reported by Angarica et al. and the weights found in the pRANK genome-wide screen. Cysteine has the lowest negative weight log-odds ratio, while its pRANK weight is close to zero. This unexpected difference has support in experiments performed by Krishnan and Lindquist [44]. They introduced a cysteine at 32 different positions in Sup35, and showed that none of these mutations affected prion activity. Another amino acid where a big difference is observed is tryptophan: it has the second lowest negative log-odds value, but is assigned a weight that is close to zero in the genome-wide pRANK evaluation. There is experimental support for the non-deleterious effect of tryptophan on prion formation: MacLea et al. showed that replacing tyrosines in Sup35 with tryptophan increases prion formation [45], and similar results were reported by Ohhashi et al. [46]. Although tryptophan is known to strongly promote aggregation, it has been suggested that it too strongly promotes prion formation, and therefore is not well tolerated in disordered segments [22]; this observation is in agreement with the analysis of Buck et al. [47]. Finally, when comparing the weights assigned by these methods with the residue propensities in protein-carbohydrate, protein-ligand, and protein-DNA binding sites computed by Malik and Ahmad [48], no obvious patterns emerge; the interested reader is referred to Fig 2 in that paper.

### In-silico mutation analysis

Paul et al. recently conducted a targeted mutation analysis of four prion-like domains from the Alberti dataset that had no detectable prion activity [32]. Four to seven prion inhibiting amino acids in each domain were replaced with either neutral or prion-promoting residues to increase the PAPA scores of the prion domain. These rationally-designed mutations led to the emergence of prion activity in two out of the four prion-like domains from Puf4 and YLR177W when inserted in place of the Sup35 prion domain. A third, from Pdc2, exhibited *in vivo* aggregation, and appeared to form unstable prions. The fourth, Yck1, formed *in vivo* aggregates but could not be tested in the prion activity assay. We performed an analogous experiment *in silico* with the aim of seeing how closely the scores of pRANK and PrionW capture these experimental findings. The results in Table 4 show that the scores of all the methods (pRANK, PAPA and PrionW) for the mutated proteins are higher than their wild-type versions (this is so for PAPA by construction). In order to classify a protein as a prion we selected a threshold score for each method. For PAPA and pRANK, this threshold is the score of the highest scoring non-prion in the Alberti dataset. For PrionW, the default score of 73.55 is used. At these threshold values, pRANK is able to correctly identify the formation of prions due to mutations for three out of the four proteins. Although *Pdc*2^*mut*^ only formed unstable prions, all three methods scored it as prion-positive. In conclusion, pRANK scores show the highest agreement with the experimental findings of Paul et al. in comparison to both PAPA and PrionW.

**Table 4:** Results of mutation analysis. Scores in bold correspond to correct predictions by a method at the given threshold value. The highest score of a non-prion in the Alberti dataset is chosen as the threshold for PAPA and pRANK whereas for PrionW, the value suggested in their paper has been used. No prion like domain was found by PrionW in *YLR*177^*W*^ and *YLR*177^*mut*^.

Protein	Effect of mutation	pRANK		PAPA		PrionW	
		WT	mutant	WT	mutant	WT	mutant
Puf4	prion formation	-0.49	**0.70**	0	**0.10**	73.85	**74.26**
YLR177W	prion formation	-1.98	**2.04**	-0.02	**0.11**	*	*
Yck1	aggregation	-4.44	**-1.74**	-0.09	0.11	64.05	82.69
Pdc2	unstable prions	0.12	**2.10**	-0.02	**0.10**	68.65	**77.18**
threshold:		0.2		0.09		73.55	

### The drivers of prion activity

The fact that we can very accurately predict prion formation based solely on amino acid composition is not contradictory to the idea that primary sequence and local regions have a role in determining prion activity. There are three basic explanations that could explain our result: 1) Local regions and primary sequence elements are not important, and only composition matters; 2) local regions and primary sequence elements are important, but the sequence requirements for these are sufficiently flexible that these elements will turn up by chance in any sequence of proper composition, and thus, their presence has little predictive value; or 3) local sequence elements do play a role in the process, but these elements can only create prions when embedded in the proper compositional context, so overall composition ends up being a more dominant factor in prediction. It is important to note that we are not arguing for the first option, and we believe that some combination of (2) and (3) holds true.

As an example, consider steric zippers predicted by ZipperDB [49]. The presence of predicted zipper segments has no predictive value for the Alberti dataset, since every single one of these proteins has predicted zipper segments [50]. So, it is entirely possible that zipper segments are necessary for prion formation and act as key nucleating sites, but because such segments are present in every prion-like domain, it is not necessary to consider them when making predictions. This idea is also strongly supported by experimental data. Deletion analysis of scrambled versions of Sup35 [22] and Ure2 [10] show that these domains each contain multiple distinct segments capable of nucleating prion formation, suggesting that nucleating sites will commonly occur by chance in domains with prion-like composition. Likewise, recent analysis of the prion domains of Sup35, Mot3, Swi1, and Ure2 identified pWALTZ segments in each protein that are capable of nucleating prion formation [51]. However, for Sup35 [52] and Swi1 [53], the identified pWALTZ segments are dispensable for prion nucleation. This suggests that, consistent with previous analysis of Sup35 [54], multiple distinct sites in these proteins can act as nucleating elements.

Another point we would like to make is that the fact that many point mutations cause pathological phenotypes is not in contradiction to our results. If a protein has an aggregation propensity that is near the threshold for aggregation, it would be expected that many different point mutations could push the protein over the edge, whether aggregation is driven by short stretches or overall composition. Indeed, we have shown that we can predict the effects of many such mutations in prion-like domains solely based on composition [32, 55].

## Methods

### Data and pre-processing

We have utilized two different datasets for this work as discussed below.

#### The Alberti dataset

The dataset generated by Alberti et al. is used as the basis for evaluating our classifiers [7]. This dataset contains 100 proteins that were tested experimentally for prion formation using four assays. A total of 18 proteins passed all four assays and are taken as the prion (positive) set. The prion domain in these 18 proteins was also localized by Alberti et al. However, no effort was made to localize the minimal fragment in these proteins that supports prion formation. Eighteen proteins failed all four assays and are taken as the non-prion (negative) set. Other than these 36 proteins, the remaining 64 proteins either showed varying levels of activities in the assays or could not be experimentally tested. Three of these proteins (Cyc8, Mot3 and Nup100) were shown elsewhere to support prion formation in their native contexts together with another protein, SFP1, which was not tested by Alberti et al. [56]. We use these four prions together with the 18 found by Alberti et al. in our analysis. We have verified that the dataset is non-redundant at the 40% sequence identity level using CD-HIT [57] by checking that no cluster contains more than positive example (prion-forming) or more than one negative example (non prion-forming). CD-HIT was run using the command line arguments -c 0.4 -n 2 -g 1. Furthermore, we also verified that the prion-forming domains are non-redundant at the 50% sequence identity level.

#### Proteome-wide datasets

In order to compare the methods at the proteome level, we created a non-redundant set of *S. cerevisiae* and human proteins. For the positive examples in yeast we used the same set of non-redundant yeast prion proteins described above; for the human dataset we used a set of seven known human prion-like proteins (TAF15, EWSR1, hnRNPA1, FUS, hnRNPA2, TDP43, and TIA1) [39]. For the construction of a non-redundant negative set, all proteins with more than 90% sequence identity to known prions were removed. The remaining proteins were then clustered based on their sequence identity using CD-HIT such that no two sequences within a cluster have more than 40% sequence identity [57]. The representative sequences of the clusters reported by CD-HIT were then used as the non-redundant negative set containing 5,575 proteins in yeast, and 15,948 in human. CD-HIT was used with the same command line as above. Non-redundancy filtering yielded five human prion-like proteins (TAF15, EWSR1, hnRNPA1, TDP43, and TIA1). Very few proteins were removed as being too similar to the positive examples. For example, in the yeast data, we started with 6,528 proteins; the first stage of filtering on the basis of similarity to yeast prions left us with 6,506 proteins, and the second step of creating a non-redundant set at the 40% sequence identity level yielded 5,575 proteins.

### Evaluation protocols

We evaluate our classifiers on the Alberti dataset to explore their specificity in detecting priogenicity in proteins that are Q/N rich and proteome-wide to determine the accuracy of proteome wide searches. All the methods we evaluated assign a score to a sequence window that reflects its priogenicity; scores are assigned at the protein level by computing the maximum score over all sequence windows within the protein.

#### Evaluation on the Alberti dataset

Evalution on the Alberti dataset was performed using a leave-one-protein-out cross-validation protocol. For this purpose, we defined a positive training set composed of 22 domains including the 18 prion-like domains that passed all four Albert et al. assays and four other known yeast prions (Cyc8, Sfp1, Mot3, and Nup100). The negative set consists of the 18 proteins that failed all four assays. In this protocol, a single protein is held-out while the classifier is trained over the set of remaining proteins. Testing is performed on the held-out protein. This process is repeated for each protein followed by calculation of the performance metrics described below.

#### Proteome-wide evaluation

We performed a proteome-wide evaluation using a bootstrap protocol as described next. For a given proteome, the set of positive examples is defined as the set of all known prion proteins in that proteome and the set of negative examples is the set of all non-redundant proteins in that proteome that are not known to have prion activity. In this protocol a prion protein is held-out while the classifier is trained on the remaining prions and a randomly selected subset of the negative proteins. The classifier is then tested on the held-out prion and the rest of the negative examples that have not been used in training. This is repeated over all prion proteins, giving classification results for all prions and a random set of negative examples. This procedure is repeated a number of times to construct an averaged ROC curve for each proteome. The ensemble of classifiers is then used to generate the predictions for a given proteome, where for a given protein only classifiers where that example has not participated as a negative example are used.

### Prediction quality metrics

In order to assess the quality of predictions, we use the following metrics:

- Area under the Receiver Operating Characteristic Curve (AUC-ROC). The Receiver Operating Characteristic (ROC) curve for a classifier is obtained through cross-validation or boot-strapping using a labeled dataset of prion and non-prion proteins. It is a plot of the true positive rate vs. the false positive rate for varying thresholds on the classification scores.
- Area under the Precision-Recall Curve (AUC-PR). For proteome level evaluation, where the number of negative examples is very large in comparison to the true positives, ROC curves are less informative. Therefore we also used Precision-Recall (PR) curves. A PR-curve is a plot of precision against recall for varying thresholds of the classification score. The area under the PR-curve (AUC-PR) is equal to 100% for an ideal classifier.

### Classifiers

**Fig 4:**
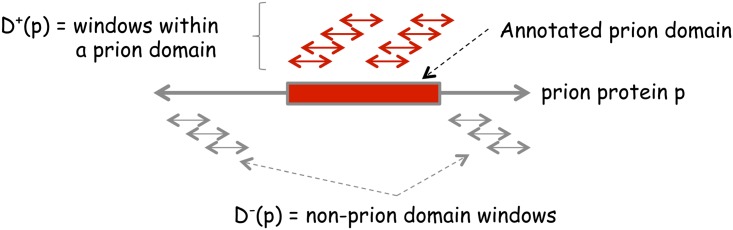
Prion prediction as a classification problem. Sequence windows within the protein are denoted by double arrows. Sequence windows within the prion-forming domain (highlighted as a red box), are in red; sequence windows that do not overlap the annotated domain are shown in grey; those are used as negative examples.

Classification of whether a given residue of a protein is within a prion domain or not is performed on the basis of features derived from residues in a sequence window centered at that residue (see Fig 4). Next, we introduce some notation used in describing our methods. We consider a labeled dataset consisting of *N* training examples {(***x***_*i*_, *y*_*i*_)|*i* = 1, …, *N*}, where ***x***_*i*_ is the feature representation of the sequence window centered at a given residue and *y*_*i*_ ∈ {+1, −1} is its associated label indicating whether the central residue of that window lies in an annotated prion domain or not. We denote by P the set of all prion proteins in the training set, and by N, the set of non-prion proteins in the training set. For a prion protein p∈P, we denote the set of fixed-size sequence windows in its annotated prion domain by *D*^+^(*p*) and the rest as *D*^−^(*p*). For a non-prion protein, all windows are labeled negative. The set of all negative examples, $N_{win}$ is defined as the collection of all sequence windows from all non-prion proteins together with the non-prion domain regions from prion proteins. Similarly, we denote the collection of all known positive examples from the prion domains of all prion proteins as $P_{win}$. This training data is used to train a classifier which can be used to score the priogenicity of a sequence window. The maximum scoring window in a protein is used for prion classification. We have considered the following classifiers in this work.

#### pWALTZ-like scoring

To evaluate whether pWALTZ accuracy is the result of primary sequence information we re-implemented the aspect of the method that uses hexamers that promote/inhibit amyloid fiber formation. A multiple sequence alignment of all those hexamers was constructed, and for each column in the alignment we computed the log-odds ratio of each residue in the two groups of amyloid promoting/inhibiting. To remove primary sequence dependency of the method we either scrambled the positions of the PSSM (equivalent to scrambling the columns of the multiple sequence alignment), or scrambled the hexamers that make up the alignment. When scrambling the hexamers, we considered 100 different permutations. A final experiment considered scrambled versions of the annotated prion domain in training/testing; for this experiment 100 permutations of each domain were used. Our implementation of pWALTZ is provided as part of the software package associated with this publication.

#### Support vector machines

As a baseline classifier, we used a linear SVM, which is commonly used in bioinformatics [35]. The SVM uses all examples in $P_{win}$ as positive examples and all examples in $N_{win}$ as negative examples. For training and evaluation of the SVM, we used the PyML package [35]. The margin violation parameter for the SVM (*C*) was coarsely optimized through cross-validation; class-specific values of *C* inversely proportional to class size were used to address class imbalance, which is the default behavior of PyML.

#### Multiple instance SVM

Multiple instance learning [33] is a supervised learning framework where examples come in bags, either positive or negative. As described earlier, in a positive bag (a prion-forming domain) it is assumed that at least one example in the bag is indeed positive; in a negative bag all examples are negative (see Fig 1). This is a weaker form of labeling positive examples which captures very well the scenario in this classification problem, where not all of the sequence windows within a bag are prionogenic. This allows the classifier to essentially ignore the windows within a bag that do not represent the target concept well. Moreover, it allows us to learn from prion proteins for which domain annotations are unavailable. This can be done by taking *D*^+^ (*p*) to be the whole protein and *D*^−^ (p) = Ø for such cases. In our problem, we model the examples in an annotated prion domain *D*^+^ (*p*) for p∈P as a single positive bag. The multiple instance SVM (miSVM) is an MIL formulation based on support vector machine that is defined as follows [33]:

$$\begin{array}{c} \begin{array}{cc} \min_{y\in {\left\{-1,+1\right\}}^{N}} & \left(\min_{w,\rho ,\xi \geq 0}\frac{1}{2}w^{T}w+\frac{C}{N}\sum_{i=1}^{N}\xi_{i}\right)\\ \text{subject}\;\text{to:} & \\ & y_{i}\left(w^{T}x_{i}+\rho \right)\geq 1-\xi_{i},\forall i\\ & \sum_{i\in D^{+}\left(p\right)}\frac{y_{i}+1}{2}\geq 1,\forall p\in P\\ & y_{i}=-1,\forall i\in N_{win}. \end{array} \end{array}$$ (1)

In this formulation, the objective is to find the optimal labeling of the examples that comprise the positive bags such that at least one example in each positive bag is labeled as positive. This is mathematically represented by the constraint $\sum_{i\in D^{+}\left(p\right)}\frac{y_{i}+1}{2}\geq 1$. The other constraints ensure correct labeling of the given training examples, and that all negative examples are labeled as negative. In our context, this means that a trained miSVM will choose at least one positive example in every annotated prion domain. All the sequence windows from the rest of the prion protein and from non-prion proteins are taken as negative examples. The miSVM formulation is a combinatorial optimization problem. We use the heuristic algorithm proposed by Andrews et al. [33] for its solution through PyML.

#### pRANK

In pRANK we aim to find a weight vector ***w*** that provides a large margin between the score of the most prion-like window in an annotated prion domain and the score of the highest scoring negative example:

$$\begin{array}{c} \begin{array}{cc} \min_{w,\xi } & \left(\frac{\lambda }{2}w^{T}w+\frac{1}{|P|}\sum_{p\in P}\xi_{p}\right)\\ \text{subject}\;\text{to:}\; & \\ & \max_{i\in D^{+}\left(p\right)}\left(w^{T}x_{i}\right)\geq \max_{j\in N_{win}}\left(w^{T}x_{j}\right)+1-\xi_{p}\;\forall p\in P\\ & \xi_{p}\geq 0\;\forall p\in P. \end{array} \end{array}$$ (2)

This formulation has several advantages over the miSVM formulation. miSVM requires optimization over the labels of examples in positive bags, making it a combinatorial optimization problem, which is solved in practice using heuristic approaches. Our formulation replaces constraints over the labels with constraints over the discriminant value of the highest scoring example in a positive bag, which is a more direct representation of the task, and lends itself to more effective optimization strategies. Furthermore, it is a simpler model with fewer slack variables, which further contributes to the speed advantage of pRANK.

To solve the pRANK constrained optimization problem presented above we proceed as follows. For a given ***w***, we define ***x***^*p*^ and ***x***^*N*^ to be the highest scoring example in *D*^+^ (*p*) and *N*_*win*_, respectively. We can then write the hinge loss corresponding to the above formulation for prion protein *p* as *l*(*p*; ***w***) = max{0, 1 − (***w***^*T*^***x***^*p*^ − ***w***^*T*^***x***^*N*^)}. Based on this definition of the loss function, we propose an approximate method inspired by the sub-gradient solver Pegasos [58]. Following the approach taken in the Pegasos paper, we first express the pRANK formulation as an unconstrained optimization problem:

$$\begin{array}{c} \min_{w}\left(\frac{\lambda }{2}w^{T}w+\frac{1}{|P|}\sum_{p\in P}l\left(p;w\right)\right). \end{array}$$ (3)

We then optimize this objective function using stochastic sub-gradient optimization. We start with a zero weight vector and at iteration *t* of the algorithm, we choose a random protein *p*_*t*_ and take a step in the direction of the sub-gradient of the objective function using the sub-gradient computed on the basis of *p*_*t*_, i.e., we consider

$$\begin{array}{c} g\left(p_{t};w\right)=\frac{\lambda }{2}w^{T}w+l\left(p_{t};w\right), \end{array}$$ (4)

whose sub-gradient is given by:

$$\begin{array}{c} g^{\prime }\left(p_{t};w_{t}\right)=\lambda w_{t}-1\left[w_{t}^{T}x^{p}<1+w_{t}^{T}x^{N}\right]\left(x^{p}-x^{N}\right). \end{array}$$ (5)

Here 1[⋅] is the indicator function which is equal to one if its argument is true (i.e., there is a margin violation) and zero otherwise, and ***x***^*p*^ and ***x***^*N*^ are the the highest scoring positive examples from the positive and negative bags, respectively. We then update the weight vector as ***w***_*t*+1_ ← ***w***_*t*_ − *η*_*t*_ *g*′ (*p*_*t*_; ***w***_*t*_) using a step size of $\eta_{t}=\frac{1}{\lambda t}$. The algorithm is stopped after a pre-determined number of iterations and the final weight vector is used for classification. Note that, at each iteration, ***x***^*p*^ and ***x***^*N*^ can change. Together with the randomness in the order of proteins, the selection of ***x***^*p*^ and ***x***^*N*^ can cause different runs of the algorithm to converge to different weight vectors. In order to limit this effect, we combine the outputs of an ensemble of 10 classifiers to produce the final prediction. The output of each classifier in the ensemble is first normalized to have the same range and then the average value of the prediction scores from different classifiers is taken as the final prediction score. An implementation of pRANK is provided as part of the software package associated with this publication.

#### Feature representation

As discussed above, our training examples are fixed-size windows; each window was represented by its amino acid composition: a vector of counts of how many times each amino acid occurs within a sequence window. In the context of SVM classifiers it is also known as the 1-spectrum feature representation [59]. Following Toombs et al. [50], we used 41 as the window size.

## Supporting information

S1 File Supplementary tables and figures. We provide results for pWALTZ like scoring without pre-filtering, ROC and PR curves corresponding to results provided in Tables 1 and 2, and detailed GO enrichment results.
(PDF)
