# Supplementary material for: Amino acid composition predicts prion activity
Source: PLoS Comput Biol. 2017 Apr 10;13(4):e1005465. doi: 10.1371/journal.pcbi.1005465 (PMC5402983; doi:10.1371/journal.pcbi.1005465)
Supplement: S1 File — We provide results for pWALTZ like scoring without pre-filtering, ROC and PR curves corresponding to results provided in Tables 1 and 2, and detailed GO enrichment results. (PDF) [file pcbi.1005465.s001.pdf]

# Amino Acid Composition Predicts Prion Activity

# Supplementary Information


### Fayyaz ul Amir Afsar Minhas, Eric D. Ross, and Asa Ben-Hur


## Accuracy of pWALTZ-like scoring without pre-filtering

In Table 1 in the paper we provided results on pWALTZ-like scoring with pre-filtering using FoldIndex and Q/N composition. In the Table below we provide additional results without pre-filtering. This experiment was performed only on the Alberti dataset, since pWALTZ-like scoring is designed to be accurate only for Q/N rich proteins.

| **PSSM type** | **AUC-ROC** | **AUC-PR** |
|---|---|---|
| original PSSM | 79.0 | 69.0 |
| scrambled PSSM | 98.0 | 98.1 |
| scrambled hexamers | 96.2 | 96.8 |
| Scrambled domains | 81.8 | 70.4 |

Table A: Results of pWALTZ-like scoring using only the PSSM. Accuracy is measured using the area under the ROC curve (AUC-ROC), and area under the precision-recall curve (AUC-PR).

## Supplemental ROC and PR curves

The figures below provide ROC and PR curves that correspond to the results reported in Tables 1 and 2 in the paper.

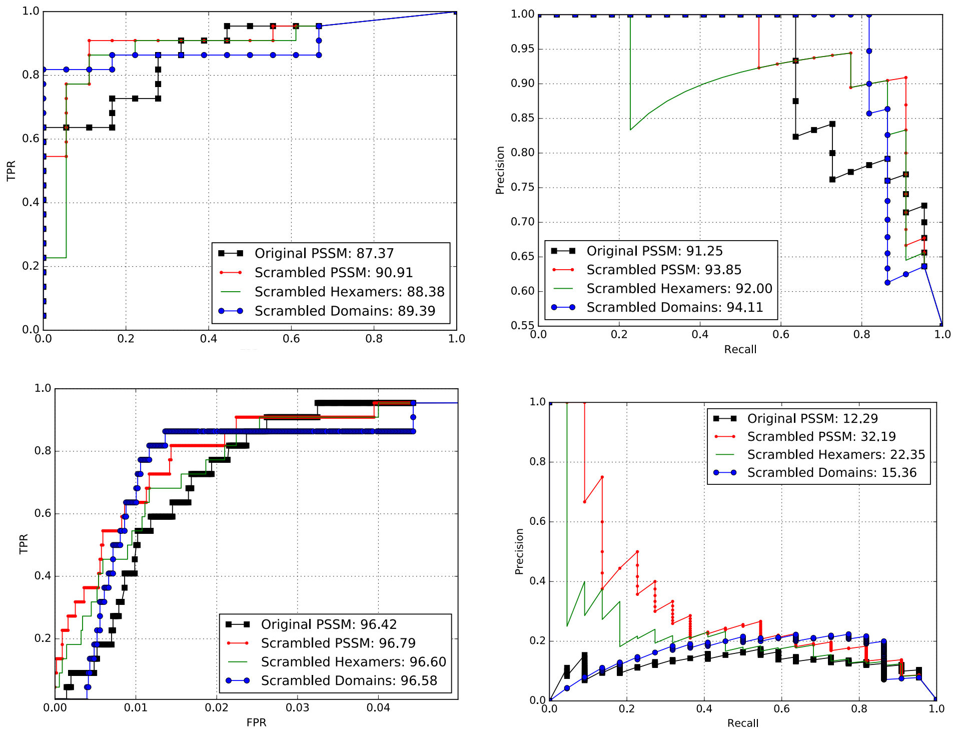


## Figure A: ROC and PR curves corresponding to Table 1.

This figures provides the ROC curves (left) and PR curves (right) on the Alberti dataset (top) and the full yeast proteome (bottom) that correspond to the results provided in Table 1 in the paper.

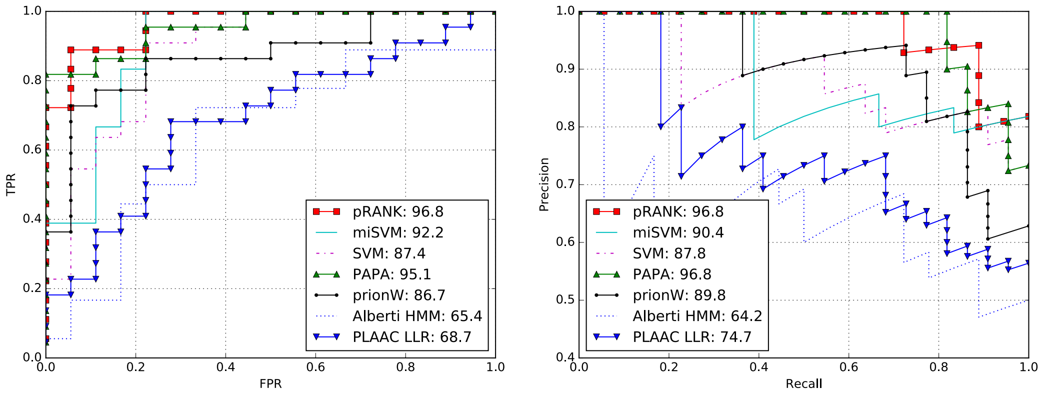


## Figure B: ROC and PR curves corresponding to Table 2.

This figures provides the ROC curves (left) and PR curves (right) on the Alberti dataset that correspond to the results provided in Table 1 in the paper.

## Gene Ontology Results

Table B: GO molecular function reported by GOrilla for the 22 yeast prion proteins:

| GO Term | Description | P-value | FDR q-value |
|---|---|---|---|
| GO:0003676 | nucleic acid binding | 1.98E-05 | 4.65E-02 |
| GO:0005487 | nucleocytoplasmic transporter activity | 2.92E-05 | 3.43E-02 |
| GO:0003729 | mRNA binding | 4.85E-04 | 3.80E-01 |
| GO:1901363 | heterocyclic compound binding | 6.39E-04 | 3.75E-01 |
| GO:0097159 | organic cyclic compound binding | 6.87E-04 | 3.23E-01 |
| GO:0001085 | RNA polymerase II transcription factor binding | 8.82E-04 | 3.46E-01 |

Table C: GO molecular function reported by GOrilla for the top 200 predictions made by pRANK:

| GO Term | Description | P-value | FDR q-value |
|---|---|---|---|
| GO:0001071 | nucleic acid binding transcription factor activity | 1.23E-19 | 2.89E-16 |
| GO:0003700 | transcription factor activity, sequence-specific DNA binding | 1.23E-19 | 1.44E-16 |
| GO:0000981 | RNA polymerase II transcription factor activity, sequence-specific DNA binding | 2.01E-19 | 1.57E-16 |
| GO:0043565 | sequence-specific DNA binding | 3.78E-13 | 2.22E-10 |
| GO:0001228 | transcriptional activator activity, RNA polymerase II transcription regulatory region sequence-specific binding | 2.71E-11 | 1.28E-08 |
| GO:0001077 | transcriptional activator activity, RNA polymerase II core promoter proximal region sequence-specific binding | 3.68E-11 | 1.44E-08 |
| GO:0003729 | mRNA binding | 1.11E-10 | 3.74E-08 |
| GO:0000982 | transcription factor activity, RNA polymerase II core promoter proximal region sequence-specific binding | 1.93E-10 | 5.66E-08 |
| GO:0001085 | RNA polymerase II transcription factor binding | 2.54E-10 | 6.63E-08 |
| GO:0003676 | nucleic acid binding | 1.82E-09 | 4.28E-07 |
| GO:0000976 | transcription regulatory region sequence-specific DNA binding | 2.67E-08 | 5.70E-06 |
| GO:0005488 | binding | 4.05E-08 | 7.94E-06 |
| GO:0044822 | poly(A) RNA binding | 7.20E-08 | 1.30E-05 |
| GO:0008134 | transcription factor binding | 9.82E-08 | 1.65E-05 |
| GO:1901363 | heterocyclic compound binding | 1.04E-07 | 1.64E-05 |
| GO:1990837 | sequence-specific double-stranded DNA binding | 1.21E-07 | 1.78E-05 |
| GO:0001067 | regulatory region nucleic acid binding | 1.29E-07 | 1.79E-05 |
| GO:0097159 | organic cyclic compound binding | 1.46E-07 | 1.91E-05 |
| GO:0000977 | RNA polymerase II regulatory region sequence-specific DNA binding | 1.69E-07 | 2.09E-05 |
| GO:0001102 | RNA polymerase II activating transcription factor binding | 2.82E-07 | 3.32E-05 |

| | | | |
|---|---|---|---|
| GO:0001012 | RNA polymerase II regulatory region DNA binding | 3.39E-07 | 3.79E-05 |
| GO:0033613 | activating transcription factor binding | 4.30E-07 | 4.60E-05 |
| GO:0000975 | regulatory region DNA binding | 5.32E-07 | 5.44E-05 |
| GO:0044212 | transcription regulatory region DNA binding | 5.32E-07 | 5.22E-05 |
| GO:0001076 | transcription factor activity, RNA polymerase II transcription factor binding | 6.82E-07 | 6.41E-05 |
| GO:0003677 | DNA binding | 1.15E-06 | 1.04E-04 |
| GO:0000987 | core promoter proximal region sequence-specific DNA binding | 2.41E-06 | 2.10E-04 |
| GO:0001159 | core promoter proximal region DNA binding | 2.85E-06 | 2.39E-04 |
| GO:0003690 | double-stranded DNA binding | 1.01E-05 | 8.22E-04 |
| GO:0001135 | transcription factor activity, RNA polymerase II transcription factor recruiting | 1.06E-05 | 8.27E-04 |
| GO:0000978 | RNA polymerase II core promoter proximal region sequence-specific DNA binding | 1.22E-05 | 9.22E-04 |
| GO:0005515 | protein binding | 2.76E-05 | 2.03E-03 |
| GO:0001134 | transcription factor activity, transcription factor recruiting | 4.05E-05 | 2.88E-03 |
| GO:0005487 | nucleocytoplasmic transporter activity | 5.27E-05 | 3.64E-03 |
| GO:0000989 | transcription factor activity, transcription factor binding | 9.32E-05 | 6.26E-03 |
| GO:0004532 | exoribonuclease activity | 1.64E-04 | 1.07E-02 |
| GO:0016896 | exoribonuclease activity, producing 5'-phosphomonoesters | 1.64E-04 | 1.04E-02 |
| GO:0000988 | transcription factor activity, protein binding | 3.99E-04 | 2.47E-02 |
| GO:0004535 | poly(A)-specific ribonuclease activity | 4.09E-04 | 2.46E-02 |
| GO:0015616 | DNA translocase activity | 4.27E-04 | 2.51E-02 |
| GO:0008270 | zinc ion binding | 5.54E-04 | 3.18E-02 |
| GO:0001103 | RNA polymerase II repressing transcription factor binding | 5.92E-04 | 3.31E-02 |
| GO:0000175 | 3'-5'-exoribonuclease activity | 8.32E-04 | 4.55E-02 |
